# Size-specific dose estimates of adult, chest computed tomography examinations: Comparison of Chinese and updated 2017 American College of Radiology diagnostic reference levels based on the water-equivalent diameter

**DOI:** 10.1371/journal.pone.0257294

**Published:** 2021-09-13

**Authors:** Xiaoyan Hu, Jie Gou, Wei Lin, Chunhua Zou, Wenbo Li

**Affiliations:** Department of Radiology, Chengdu First People’s Hospital, Chengdu, Sichuan Province, China; Pisa University Hospital, ITALY

## Abstract

**Rationale and objectives:**

This study aimed to compare the volume computed tomography dose index (CTDIvol), dose length product (DLP), and size-specific dose estimate (SSDE), with the China and updated 2017 American College of Radiology (ACR) diagnostic reference levels (DRLs) in chest CT examinations of adults based on the water-equivalent diameter (Dw).

**Materials and methods:**

All chest CT examinations conducted without contrast administration from January 2020 to July 2020 were retrospectively included in this study. The Dw and SSDE of all examinations were calculated automatically by “teamplay”. The CTDIvol and DLP were displayed on the DICOM-structured dose report in the console based on a 32cm phantom.The differences in patient CTDIvol, DLP, and SSDE values between groups were examined by the one-way ANOVA. The differences in patient CTDIvol, DLP, and SSDE values between the updated 2017 ACR and the China DRLs were examined with one sample t-tests.

**Results:**

In total 14666 chest examinations were conducted in our study. Patients were divided into four groups based on Dw:270 (1.84%) in 15–20 cm group, 10287 (70.14%) in the 21–25 cm group, 4097 (27.94%) in the 26–30 cm group, and 12 (0.08%) patients had sizes larger than 30 cm. CTDIvol, DLP, and SSDE increased as a function of Dw (*p*<0.05). CTDIvol was smaller than SSDE among groups (*p*<0.05). The mean CTDIvol and DLP values were lower than the 25^th^, 50^th^, and 75^th^ percentile of the China DRLs (p <0.05). The CTDIvol, DLP, and SSDE were lower than the 50^th^ and 75^th^ percentiles of the updated 2017 ACR DRLs (p <0.05) among groups.

**Conclusions:**

SSDE takes into account the influence of the scanning parameters, patient size, and X-ray attenuation on the radiation dose, which can give a more realistic estimate of radiation exposure dose for patients undergoing CT examinations. Establishing hospital’s own DRL according to CTDIvol and SSDE is very important even though the radiation dose is lower than the national DRLs.

## 1. Introduction

Following the progress of medical examination technology and the need of medical diagnosis and treatment, computed tomography (CT) examinations have gained popularity for the diagnosis, differential diagnosis, and disease followups. CT is by far the most important source of radiation exposure in diagnostic radiology, and the radiation dose from CT examination accounts for a large proportion of medical radiation sources [1–3]. Previous research has showed that 70% of the radiation doses received by patients during medical treatment were attributed to CT scans [4]. Radiation damage attributed to both deterministic and stochastic effects caused by CT radiation have been reported. Despite the benefit of medical practices with ionizing radiation, more and more attention is being paid to the risk associated with such practices. Accordingly, an accurate estimate of the radiation dose is very important.

Currently, the volume CT dose index (CTDIvol, mGy) and the dose length product (DLP, mGy.cm) are the two most common parameters used to describe the radiation exposure from CT examinations [5–11]. CTDIvol was developed to provide a standardized method to compare radiation output levels between different CT scanners using a reference phantom. DLP is calculated by multiplying CTDIvol with the length of the scan volume. CTDIvol and DLP are sensitive to tube voltage, tube current, rack rotation time, pitch,and both metrics only calculated the radiation output within standard head and body phantom. However, the radiation exposure of patients depends on both the radiation output of the scanner and the patient size. Therefore, interpreting CTDIvol as a patient dose may lead to the overestimation or underestimation of the patient dose, and thus inaccurate estimates of radiation risks [12]. Until now, the diagnostic reference levels (DRLs) have been based on CTDIvol and DLP. However, there is large body size heterogeneity among different population. Therefore, it is difficult to use these DRL estimates for dose analysis and dose optimization of patient size subgroups. Owing to the limitations of CTDI_vol_ (used as a parameter for patient dose exposure), dose estimation methodologies that take into account the patient size have gained importance. The size-specific dose estimate (SSDE), was proposed by the American Association of Physicists in Medicine (AAPM) task groups 204 and 220 [13,14]. In 2011 [13] and 2014 [14], the AAPM published reports on the calibration of CTDIvol for SSDE based on the effective diameter (ED) and water-equivalent diameter (Dw) for patients who were planned to undergo CT examinations. SSDE is an alternative dose metric in close relationship with CTDIvol and takes into account the impact of both the scanning parameters and patient size variations. Recently, there has been a widespread use of SSDE as an alternative to CTDIvol [15–18]. In 2017, the American College of Radiology (ACR) updated the diagnostic references levels of CT examinations for different patient size groups based on SSDE [19].

The patient’s Dw was used to quantify patient’s size in our study. We analyzed the CTDIvol, DLP, and SSDE of the chest CT examinations in different Dw subgroups, and compared them with the China [20] and updated 2017 ACR DRLS [19] to detect the potential for CT dose optimization of patient size, to monitor radiation dose for chest CT based on body size, and to explore the importance and necessity of establishing the institutional DRLs.

## 2. Materials and methods

### 2.1 Patients

This retrospective study was approved by our hospital ethics committee and in accordance with declaration of Helsinki. The subjects included are patients who went to the institution for CT exams, so the informed consent was waived with the approval of the ethics committee of the hospital. All routine chest CT examinations from January 2020 to July 2020 were retrospectively included in this study. The exclusion criteria were as follows: 1) patients who had metal foreign bodies on body surfaces or metal fixators in their chests, 2) patients with pacemakers, 3) patients with poor cooperation during examinations, 4) patients who were re-scanned owing to operational errors or patient reasons (for instance, patient didn’t hold their breath or moved during the examination; the technician chosen the wrong scanning protocol, and so on), and 5) pediatric patients for which the ACR does not provide a reference DRL.

### 2.2 Size-specific dose estimate and data acquisition

SSDE is CTDIvol with multiplicative conversion factors (*f_size_*) which depend on Dw, as described in AAPM220[14]. SSDE and Dw were obtained through the previously validated software teamplay (Siemens Healthineers, Germany). Dw was determined by the axial CT image. The CTDIvol and DLP are displayed on the monitor and included in the DICOM-structured dose report based on a 32cm phantom in all examinations. SSDE and Dw were calculated by the software according to the AAPM 220 from the following equations:

$$SSDE=f_{size}^{32x}\times {CTDI}_{vol}^{32}$$ (1)

$$f_{size}^{32x}=a\times e^{-b\times Dw}$$ (2)

$$Dw=2\sqrt{[\frac{1}{1000}\bar{CT{\left(x,y\right)}_{ROI}}+1]\frac{A_{ROI}}{\pi }}$$ (3)

where $\bar{CT{\left(x,y\right)}_{ROI}}$ is the mean CT number in the ROI,HU; *A_ROI_* is the total area of the ROI,cm^2^; $\bar{CT{\left(x,y\right)}_{ROI}}$ and *A_ROI_* were automatically calculated by teamplay according to the axial CT image. $f_{size}^{32x}$ is conversion factors. According to the AAPM 204 [13], the coefficients a and b in Eq (2) were: a = *4*.*378*, *b = 0*.*043*.

### 2.3 Imaging protocol

CT examinations were performed with a SOMATOM Definition Flash scanner (Siemens, Germany). Automatic exposure control (care KV) and automatic tube current modulation (care dose 4D) were activated on all patients. The preset parameters were as follows: tube voltage: 80–120 kVp, quality reference mAs: 100 mAs, detector collimation: 128×0.6 mm, acquisition matrix: 512×512, field-of-view 314×314 mm, reconstruction source image slice thickness: 1 mm, and inter-slice spacing: 1 mm. Care KV and care dose 4D would adjust appropriate parameters according to patient’s size. (Table 1). All patients were placed in a supine position with the head first. The scanning range was from the lung apex to the adrenal glands. All images included in this study were rated as excellent by the reporting physician in the daily quality control evaluation.

**Table 1:** Scan parameters of subjects in each group.

Group	tube voltage(kVp)	mAs(mAs)#	pitch	slice hickness(mm)
Dw_15-20_	100(246/270,91.11%*)	88.22±21.82	1.5	1
	120(24/270,8.89%*)	77.51±12.62		
Dw_21-25_	80(2/10287,0.019%*)	130	1.5	1
	100(6025/10287,58.57%*)	98.50±19.67		
	120(4260/10287,41.41%*)	81.12±17.64		
Dw_26-30_	100(697/4097,17.01%*)	98.26±20.84	1.5	1
	120(3400/4097,82.99%*)	79.09±21.21		
Dw_>30_	120(12/12,100%*)	81.82±29.05	1.5	1

* Percentage of the corresponding tube voltage

# The average mAs of each tube voltage in each subgroup.

### 2.4 Statistical analyses

SPSS (version 22, IBM Inc., NY, USA) was used for all data analysis. The Kolmogorov–Smirnov normality test and Levene’s test for homogeneity of variance were used for all the data. *P* values < 0.05 were considered statistically significant. Mean and standard deviation were computed for the patient age, CTDIvol, DLP, and SSDE. Dw was not normally distributed, so median and interquartile range were computed for the Dw. Spearman correlation analysis was used to analyze the correlation between CTDIvol, SSDE and Dw. The differences in patient CTDIvol, DLP, and SSDE values among the groups were examined with one-way analysis of variance (ANOVA) tests. Independent-samples T test was used to compare CTDIvol and SSDE within the group.The difference in patient CTDIvol, DLP, and SSDE between the ACR and the Chinese DRLs were examined by one-Sample t-tests.

## 3. Results

### 3.1 Patient characteristics

**Fig 1:**
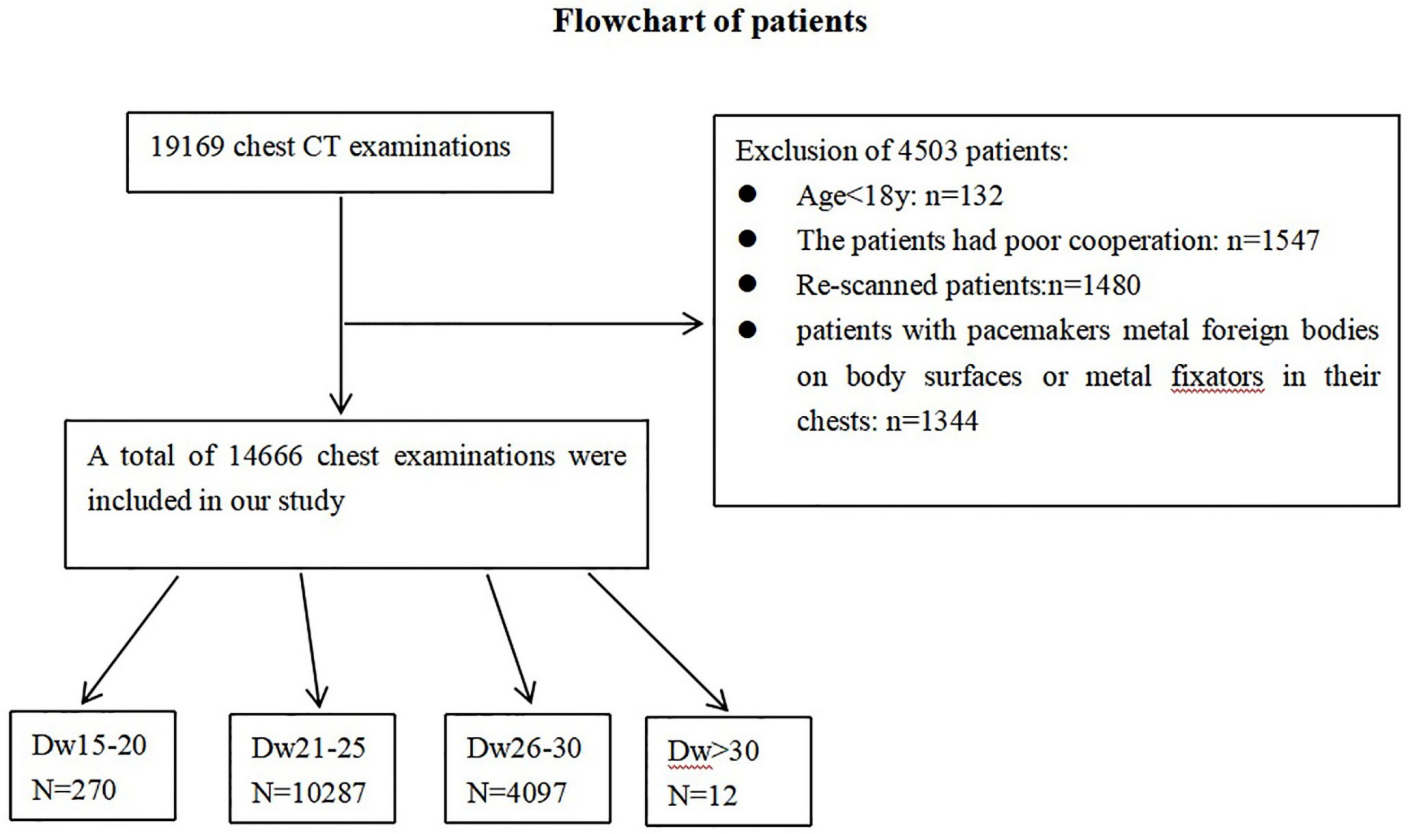
Number of CT examination enrolled in this study and divided into subgroups on the basis of water-equivalent diameter(Dw), with exclusion criteria.

In total, 19169 chest CT examinations had been conducted at our institution from January 2020 to July 2020. According to the exclusion criteria, a total of 14666 chest examinations were included in our study. These included 6322 males and 8344 females (mean patient age 46.64 ± 17.51 years). The minimum Dw was 17.42 cm, the maximum Dw was 32.29cm, and the median Dw was 23.74cm. Patients were divided into four groups based on Dw. Among the 14666 chest examinations, 270 (1.84%) fell in the 15–20 cm size range, 10287 (70.14%) fell in the 21–25 cm size range, 4097 (27.94%) fell in the 26–30 cm size range, and 12 (0.08%) patient sizes were larger than 30 cm (Fig 1, Table 2).

**Table 2:** Patient characteristics.

	Dw_15-20_	Dw_21-25_	Dw_26-30_	Dw_>30_
n	270	10287	4097	12
Dw(cm)	19.58±0.41	22.95±1.25	26.29±1.01	30.70±0.70
Sex(M, F)	53,217	4105,6182	2156,1941	8,4
Age(y)	42.28±20.11	44.25±17.44	52.78±15.90	59.5±15.49

### 3.2 Radiation dose analyses between subgroups

**Fig 2:**
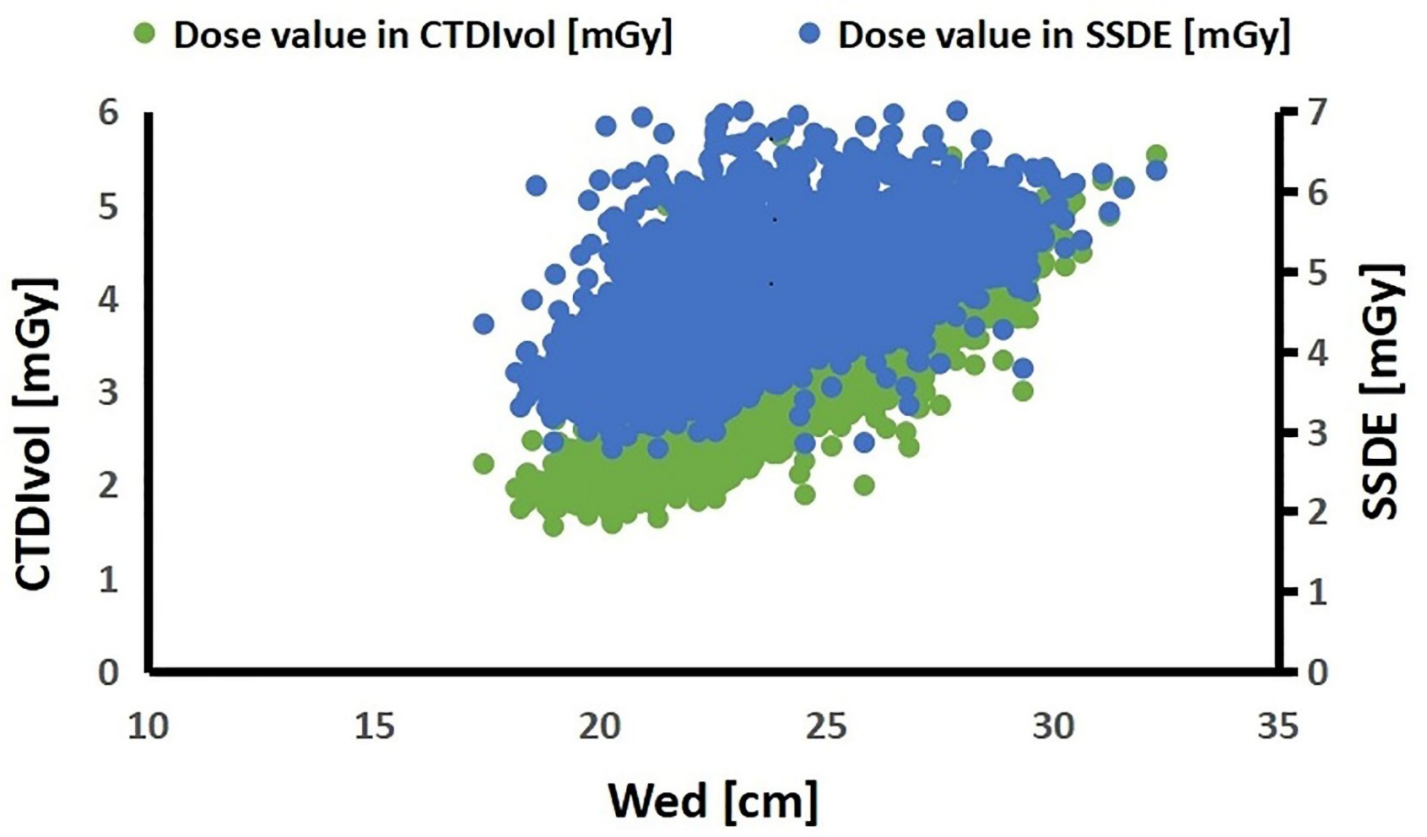
Scatter plots of CTDIvol, SSDE and Dw. The CTDIvol and SSDE increased as function of Dw.

**Fig 3:**
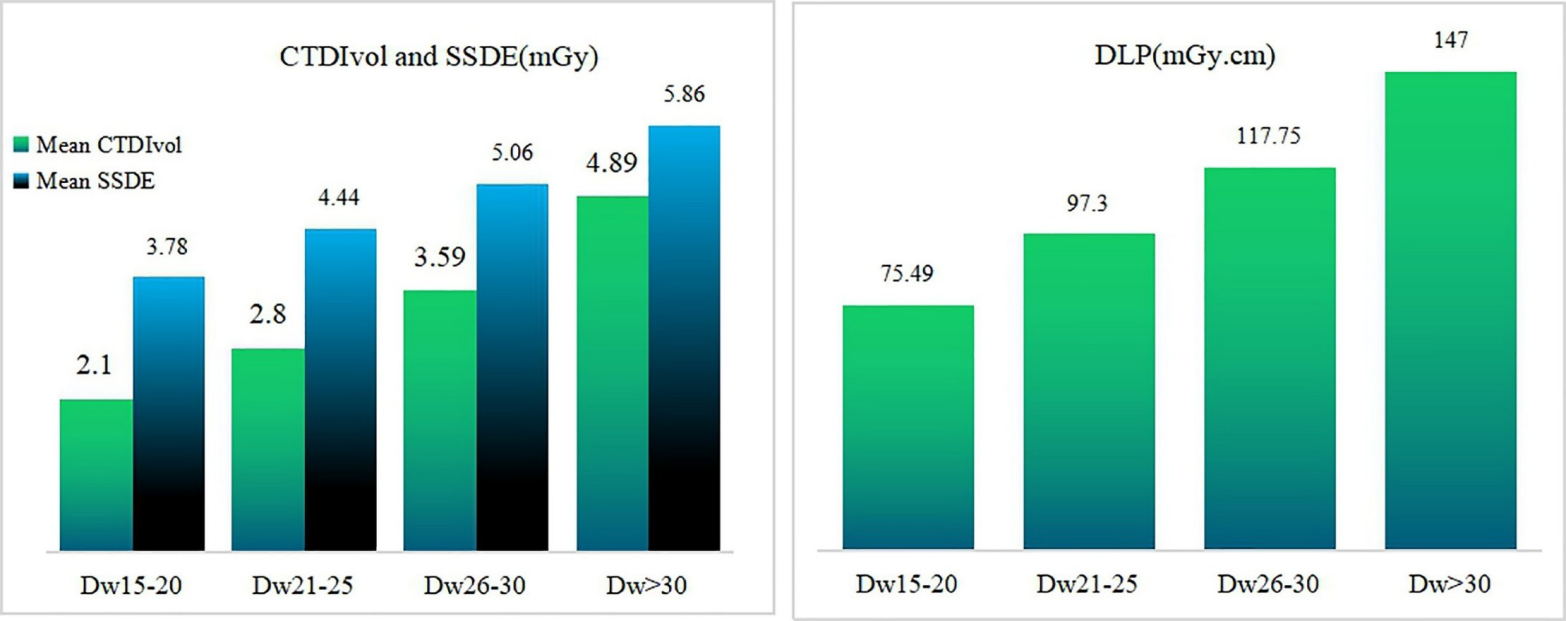
Comparison of CTDIvol, DLP, and SSDE among different size groups. The CTDIvol was smaller than SSDE in each subgroups.

Spearman correlation analysis showed that CTDIvol had a strong positive correlation with Dw (R = 0.873, *p* = 0.000), and SSDE had a positive correlation with Dw (R = 0.705, *p* = 0.000). The scatter plots of CTDIvol, SSDE as function of Dw were shown in Fig 2. The dose analysis among the studied subgroups showed that CTDIvol, DLP, and SSDE increased as a function of Dw. The differences were statistically significant. The comparison between CTDIvol and SSDE in each subgroup showed that CTDIvol was smaller than SSDE, and the difference was statistically significant (Fig 3, Table 3).

**Table 3:** CTDIvol, DLP, and SSDE analysis among subgroups, and comparison CTDIvol and SSDE between groups.

Size group	n	Mean DLP(mGy.cm)	Mean CTDIvol(mGy)	Mean SSDE(mGy)	T*	*P* *
Dw_15-20_	270	75.49±10.74	2.10±0.25	3.78±0.44	-54.97	<0.001
Dw_21-25_	10287	97.30±15.42	2.80±0.39	4.44±0.50	-258.84	<0.001
Dw_26-30_	4097	117.75±15.15	3.59±0.37	5.06±0.40	-171.98	<0.001
Dw_>30_	12	147.00±10.7	4.89±0.34	5.86±0.31	-7.28	<0.001
F	-	2074.42	4899.44	1982.03	-	-
*p*	-	<0.001	<0.001	<0.001	-	-

*: Independent-samples t test was used to compare CTDIvol and SSDE within the group. T refers to the statistical test value of Independent-sample t test. F one-way analysis of variance tests.P<0.05 was statistically significant.

### 3.3 Comparison between CTDIvol and DLP with China DRLs

For all included chest CT examinations, the mean CTDIvol was 3.01 ± 0.54 mGy, which was 50%, 62%, and 80% lower than the 25^th^ percentile, 50^th^ percentile, and 75^th^ percentile of China DRLs, respectively. The difference was statistically significant (p <0.05).The mean DLP was 102.63 ± 18.22 mGy.cm, which was 49%, 66%, and 78% lower than the 25^th^ percentile, 50^th^ percentile, and 75^th^ percentile of the China DRLs, respectively. The difference was statistically significant (p <0.05). (Table 4).

**Table 4:** Comparison between CTDIvol and DLP in our study with China DRLs.

	25^th^ percentile		50^th^ percentile		75^th^ percentile	
	CTDIvol	DLP	CTDIvol	DLP	CTDIvol	DLP
Reference value of China DRLs	6	200	8	300	15	400
Percentage*	50%	49%	62%	66%	80%	78%
T	-672.443	-647.636	-1121.996	-1311.697	-2695.026	-2441.621
*p*	<0.001	<0.001	<0.001	<0.001	<0.001	<0.001

*:Compared with DRLs of China, the CTDIvol and DLP were all lower than the 25^th^ percentile, 50^th^ percentile 75^th^ percentile DRLs of China. T refers to the statistical test value of one-Sample t-test. *P*<0.05 was statistically significant.

### 3.4 Comparison of CTDIvol, DLP, and SSDE based on the updated 2017 ACR DRLs

**Fig 4:**
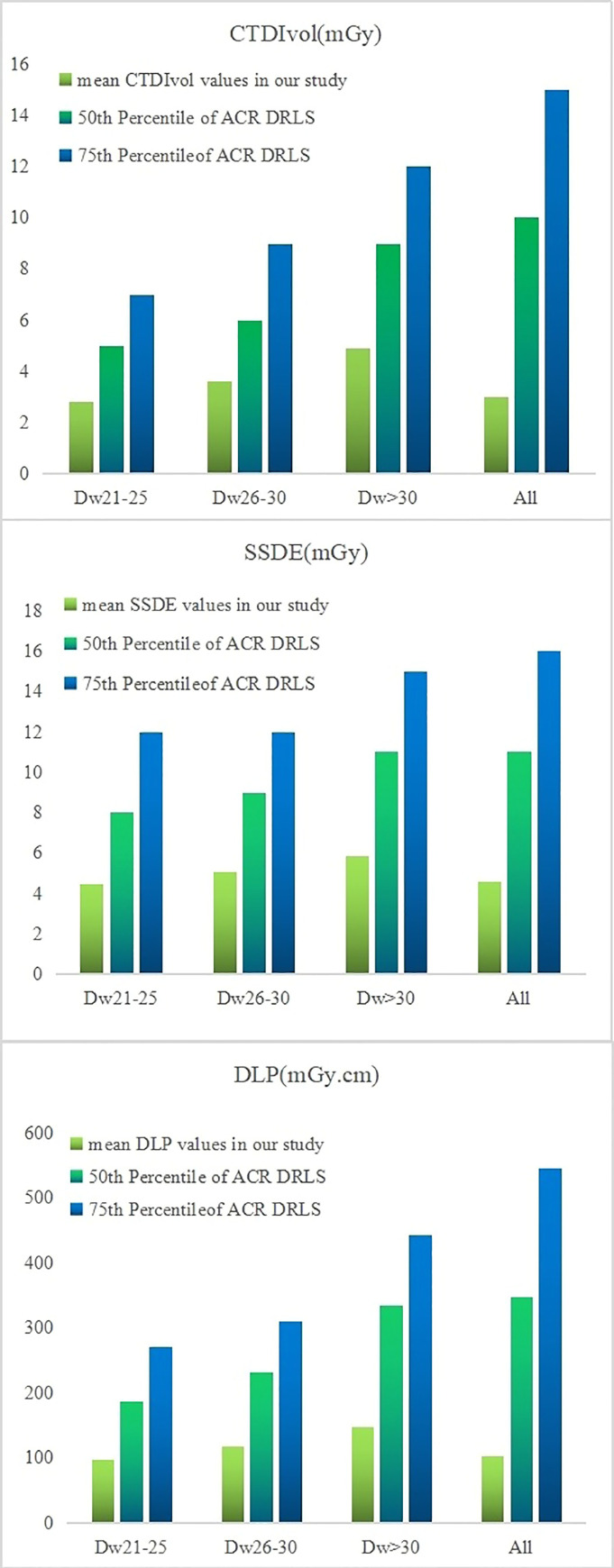
CTDIvol, DLP, and SSDE in our study were all lower than the 50^th^ and 75^th^ percentiles of the ACR DRLs among groups.

In the Dw21–25, Dw26–30, and Dw > 30 groups, the CTDIvol, DLP, and SSDE in our study were all lower than the 50^th^ and 75^th^ percentiles of the updated ACR DRLs, and the one-Sample t-tests showed that the difference were statistically significant (p <0.05) (see Fig 4, Tables 5 and 6).

**Table 5:** The mean values of CTDIvol, DLP, SSDE in our study and mean values of 50^th^ percentile and 75^th^ percentile of ACR DRLs.

	CTDIvol			SSDE			DLP		
	mean value in our study	50^th^ Percentile	75^th^ Percentile	mean value in our study	50^th^ Percentile	75^th^ Percentile	mean value in our study	50^th^ Percentile	75^th^ Percentile
Dw_21-25_	2.8	5	7	4.44	8	12	97.3	186	270
Dw_26-30_	3.59	6	9	5.06	9	12	117.75	231	310
Dw_>30_	4.89	9	12	5.86	11	15	147	334	443
All	3.01	10	15	4.6	11	16	102.63	347	545

**Table 6:** Comparison of the CTDIvol, DLP, and SSDE with the 50^th^ and 75^th^ percentiles of ACR DRLs.

	CTDIvol					
	50th Percentile			75th Percentile		
	Percentage*	T	*p*	Percentage*	T	*p*
Dw_21-25_	44%	-575.379	<0.001	75%	-1097.577	<0.001
Dw_26-30_	40%	-417.682	<0.001	60%	-938.356	<0.001
Dw_>30_	46%	-41.791	<0.001	59%	-72.282	<0.001
All	70%	-1571.369	<0.001	80%	-2695.026	<0.001
	SSDE					
	50th Percentile			75th Percentile		
	Percentage*	T	*p*	Percentage*	T	*p*
Dw_21-25_	45%	-727.512	<0.001	63%	-1545.737	<0.001
Dw_26-30_	44%	-627.337	<0.001	58%	-1104.926	<0.001
Dw_>30_	47%	-57.086	<0.001	61%	-101.505	<0.001
All	58%	-1388.969	<0.001	71%	-2474.864	<0.001
	DLP					
	50th Percentile			75th Percentile		
	Percentage*	T	*p*	Percentage*	T	*p*
Dw_21-25_	44%	-583.573	<0.001	64%	-1136.137	<0.001
Dw_26-30_	52%	-478.357	<0.001	63%	-812.031	<0.001
Dw_>30_	56%	-60.543	<0.001	67%	-95.831	<0.001
All	70%	-1624.088	<0.001	81%	-2940.117	<0.001

*:Compared with DRLs of **ACR DRLs**, the CTDIvol, SSDE and DLP were all lower than the 50^th^ percentile 75^th^ percentile DRLs of **ACR DRLs. T** refers to the statistical test value of one-Sample t-test. *P*<0.05 was statistically significant.

## 4. Discussion

AAPM task groups 204 and 220 introduced the concept of using the dimension including anterior posterior, lateral, ED, and Dw of the patient to calculate the SSDE. The AAPM task group 220 confirmed that the ED was 4.3%–21.5% greater than Dw in the thorax because the ED considered only the geometry but not the attenuation that would lead to an overestimation of the patient size and to the underestimation of SSDE. This study was about radiation dose in chest CT scans, the SSDE calculation was decided by the Dw.

The CTDIvol, DLP, and SSDE of chest CT examinations increased as the patient size increased in this study, and there were strong positive correlation for both CTDIvol and SSDE with Dw. This was expected because in this study, all subjects used automated exposure control for chest CT examination (care dose 4D and care KV). Both image quality and radiation dose depended on the size and shape of patient and scanner parameter [21]. To achieve the comparable image quality, larger patients needed higher doses. The radiation dose increased as the patient body size increased. This finding was consistent with findings reported in previous studies [22–24]. In the study which used fixed tube current (FTC) and tube current modulation (TCM) on SSDE and image quality in lung cancer screening CT, Barreto [24] demonstrated that SSDE increased exponentially as patient size increased, and all images were still deemed clinically acceptable. Furthermore, we found that the radiation exposure dose for chest CT examinations in our study was lower than China DRLs and updated 2017 ACR DRLs. This finding can also be explained by automated exposure control. A phantom study used TCM in thoracic and abdominal–pelvic imaging yielded significant radiation dose reduction [25]. Care dose 4D is an automated exposure control method that ensures consistent diagnostic image quality over all body regions at a reduced dose. Care dose 4D combined three different adaptation methods to optimize imaging quality at the lowest level: (1) automatic adaptation of the tube current to the patient size, (2) automatic adaptation of the tube current to the attenuation of the patient’s long axis, and (3) automatic adaptation of the tube current to the angular attenuation profile measured online for each single tube rotation. Care KV optimized the applied dose for the defined image quality level, and reduced the influences owing to the technical limits of the system. To achieve this, care KV adapted the tube voltage and current. All images include in the study was rated excellent to meet diagnostic requirements by respective reporting physicians in charge of quality control.

There were 14666 chest CT examinations included in our study. The water-equivalent diameter varied among patients. Most of the subjects fell in the 21-25cm size range.The proportion of small subjects was very low. Only one subject’s Dw was larger than 32cm. No too large-sized subjects were included in this study. We calculated the mean radiation doses of chest examination in all Dw subgroups and found that CTDIvol values were lower than the SSDE in all Dw groups. This finding was consistent with the findings in previous studies [19,26], which has to be expected because it reflects the correction factors provided by the AAPM 204 and 220. CTDIvol in chest CT scan is generally calculated using the phantom of 32cm. But the actual diameter of the vast majority of included subjects were less than 32cm, So the actual radiation doses were larger than CTDIvol value. According to the AAPM report 220, the conversion factor is larger than 1 for all Dw smaller than 35 cm, which can explain the reason why SSDE in this study is higher than CTDIvol. CTDIvol can only offer information about the scanner output for a standardized condition [27]. However, the radiation exposure dose of CT examination was related to the scanner output and on the patient’s geometric size and X-ray attenuation [14]. Using Monte Carlo and analytical methods, Wang et al. [28] proved that the use of Dw could estimate the dose of an object and yielded an accurate absorbed dose value. SSDE is CTDIvol with multiplicative conversion factors which depend on ED or Dw, which takes into account the influence of both the scanning parameters and patient size on the radiation dose. which can give a more realistic estimate of radiation exposure dose for patients undergoing CT examinations. In Klosterkemper’s [22] study, they found that CTDIvol was larger than SSDE when the Dw values of patients who underwent chest and abdomen CT scanning Dw was in the range of 37–43 cm. For patients with larger sizes, CTDIvol tended to overestimate the radiation dose. However, in our institution, no obese patients were included in this study, which is consistent with the shape characteristics of Asians.

Comparison of the CTDIvol, DLP, and SSDE of our institution with the China DRLs [20] and updated 2017 ACR DRLs [19], we found that the radiation doses among the subgroups were all lower than the China and ACR DRLs. DRLs were defined as a survey level that applies to an easily measured quantity using a standard phantom or a patient with a representative size [19]. Therefore, it may not be suitable for too small or too large patients. More importantly, DRLs are established regionally and nationally, which are dose distribution derived from a survey conducted on a wide user base (including large, small, public, private, hospital, and outpatient facilities) and different manufacturers and models of equipment, and varied extensively among regions and countries [29,30]. Our institution is located at the southwest part of China, where the majority of the patient population has body sizes less than national average. Taking into account the skewness of the patient population, it is understandable that most of the recorded radiation doses experienced by patients in this study were lower than the national DRL. Comparing our institution radiation dose with the DRLs of China and ACR, although no significant high-dose events have been observed, if the radiation dose is abnormally higher than the average radiation dose of our institution, we still need to examine the medical procedures and equipment to see if there are any problems, and need to establish robust dose protocols to ensure necessary measures (such as personnel training, equipment check, component replacement, etc) be taken immediately once such events occur. In practical work, threshold values can be set according to CTDIvol and SSDE dose reference levels collected by our own hospital. When the system suggests that the radiation dose of a subject exceeds the threshold, the application of the scanning parameters of the examination should be traced in time to allow for potential corrections in subsequent examinations, aiming to reduce probability of radiation damage caused by malpractice of the scanning protocol. It makes more sense to be specific to subgroups. Based on the “as low as reasonably achievable” principle [31], the radiation dose is required to be as low as possible on the premise of ensuring sufficient diagnostic image quality and diagnostic accuracy. Therefore, according to the patient size distribution and dose characteristics in our institution, it is inferred that establishing our institution DRLs will be more favorable to the radiation dose supervision and management of the patients. However, we did not use this study to establish the DRL of our hospital for the various subgroups, because we believed that the sample size of this study was far from sufficient to establish a reasonable and applicable DRL. Moreover, only one type of CT device was included in this study, and the results cannot be generalized to other types of CT devices. By further increasing the sample, increasing the type of equipment, and combining several hospitals in the region, the more accurate and reasonable DRLs based on SSDE were established for hospitals and regions, which is something we want to study further.

There are limitations associated with our study. Firstly, only chest CT without contrast material examinations were included in this study. We did not analyze the radiation dose characteristics of other examinations, such as the abdomen, head, spine, limbs, etc. Secondly, although the radiation dose of the chest CT examinations in our institution was lower than the DRLs of China and the 2017 ACR DRLs, we did not analyze the image quality individually. All images involved in this study were rated excellent for diagnosis by reporting physicians, therefore at least we have confidence there was no clear quality problem encountered. Thirdly, no large-sized subjects were included in our study. Although obese population among Asian groups tends to be under-represented, it is still important to set up dose protocols for over-sized patients. Therefore, in clinical work, in the case of obese patients, the scanning parameters should be appropriately adjusted to maintain the optimal radiation dose and image quality.

## 5. Conclusions

In conclusion, SSDE takes into account the influence of the scanning parameters, patient size, and X-ray attenuation on the radiation dose, which can give a more realistic estimate of radiation dose for patients undergoing CT examinations and is less likely to underestimate patient radiation dose compared with conventional CTDIvol for small-sized patients. DRLs are only presentative of the average dose standards across the country. For regions with patient size significantly smaller than national standards, such DRLs may not be suitable. Blindly following the national standards will potentially lead to relaxation of the correct dose levels and thus negligence of harmful dose events. Especially for a country of population of nearly 1.4 billion, the discrepancy could be larger than expected. Therefore, one should be extremely cautious of abnormal dose events and make routine equipment checks to minimize the likelihood of potential harmful radiation dose events. In the meantime, establishing a suitable institutional standard based on current practice is equally important.

## Supporting information

S1 Dataset Raw data for the study. (XLS)
